# How well do documented goals-of-care discussions for patients with stage IV cancer reflect communication best practices?

**DOI:** 10.1186/s12904-021-00733-2

**Published:** 2021-03-10

**Authors:** Natalie C. Ernecoff, Kathryn L. Wessell, William A. Wood, Gary S. Winzelberg, Frances A. Collichio, Laura C. Hanson

**Affiliations:** 1 Department of Medicine, Division of General Internal Medicine, University of Pittsburgh School of Medicine, 3600 Forbes Avenue, 405.35 Iroquois Building, Pittsburgh, PA 15213 USA; 2 Sheps Center for Health Services Research, University of North Carolina, Chapel Hill, USA; 3 Department of Medicine, Division of Hematology/Oncology, University of North Carolina, Chapel Hill, USA; 4 Department of Medicine, Division of Geriatric Medicine and Palliative Care Program, University of North Carolina, Chapel Hill, USA

**Keywords:** Metastatic, Decision making, Palliative care, Electronic health record

## Abstract

**Background:**

Written clinical communication regarding patients’ disease understanding and values may facilitate goal-concordant care, yet little is known about the quality of electronic health record (EHR) documentation. We sought to (1) describe frequency of communication best practices in EHR-documented goals-of-care discussions, and (2) assess whether templated notes improve quality of documentation.

**Methods:**

Researchers pulled text of EHR-documented goals-of-care discussions for hospitalized patients with Stage IV cancer from admission to 60-days follow-up. Text was included when in a single encounter the clinician addressed: (a) prognosis and/or illness understanding; and (b) goals and/or treatment options. Researchers qualitatively coded text based on guidelines for communication best practices, and noted if an EHR template was used.

**Results:**

Forty-two percent (206/492) of patients had EHR-documented goals-of-care discussions. Text frequently described communication of cancer progression (89%), though rarely included prognosis (22%). Text often included patients’ goals and values (83%), and at least on specific treatment decision (82%). Communication about treatments was included for 98% of patients; common examples included cancer treatment (62%), hospice (62%), resuscitation (51%), or intensive care (38%). Clinicians documented making recommendations for 40% of patients. Text addressing patient emotional and spiritual concerns was uncommon (15%). Compared to free text, use of a template was associated with increased documentation of goals and values (80% vs. 61%, *p* < 0.01), but not other best practices.

**Conclusion:**

Insights from the study can be used to guide future training and research to study and improve the quality of documentation about goal of care, and its impact on goal-concordant care.

## Background

### Background

High-quality care for patients with serious illness requires skilled goals-of-care communication in order to be concordant with patients’ goals, values and preferences [1, 2]. Observational research of in-person communication has helped define best practices for communication and decision-making [3, 4]. Communication skills training has demonstrated capacity to improve skills for in-person clinician-patient communication [5–7]. While high-quality clinician-patient communication is important; written evidence of this communication in the health record may promote delivery of goal-concordant care. For example, in hospital written records of patients’ goals and values, key treatment decisions are used by clinicians covering at night or on weekends to guide treatment. Teaching best practices for in-person communication is not sufficient to ensure high quality documentation [8].

Providing goal-concordant care, a key outcome to improve serious-illness care, includes communication between clinicians in the form of high-quality documentation of goals-of-care discussion in the electronic health record (EHR) [9]. The content of high-quality documentation may slightly differ from high-quality communication; for example, whether decisions were made and—perhaps more important for future decision-making communication—what goals and values the patient expressed to guide their choices. Very limited research explores how well goals-of-care communication is documented in the EHR, but existing evidence indicates it is insufficient [10–13]. Understanding how current documentation reflects communication best practices may be a first-step toward use of EHR documentation as a tool to promote goal-concordant care.

In this study of hospitalized patients with Stage IV cancer, we sought [1] to describe frequency of communication best practices in EHR-documented goals-of-care discussions; and [2] to assess whether templated notes for Advance Care Planning (ACP) improve quality of documentation [14].

## Methods

### Study design

We abstracted (KLW and two other chart abstractors) and qualitatively coded (NCE, KLW) content of all documented goals-of-care discussions from clinician notes (generally oncology, palliative care, and medicine) in the EHR among hospitalized patients with Stage IV cancer. Chart reviews were part of a pre/post study of a collaborative model of oncology-palliative care among all patients with Stage IV cancer admitted to one inpatient medical oncology service at the University of North Carolina (UNC) Medical Center from June 01, 2017 to November 15, 2018. The UNC institutional review board approved all study procedures.

### Inclusion and exclusion criteria

We included all adult patients with Stage IV solid-tumor cancer who were admitted to the inpatient Medical Oncology unit with an acute illness, and who received their primary outpatient oncology care at UNC Medical Center. We excluded those with a planned admission, or who were prisoners at the time of admission, requiring a different process for decision-making.

### Data collection

#### Chart reviews

We conducted structured, systematic chart reviews from admission through 60 days post-admission date to abstract data from the inpatient and outpatient EHR (Epic) for each patient. Chart reviews included demographic and clinical characteristics, encounter details and utilization information, and elements of palliative care including pain and symptom screening and goals-of-care discussion. To ensure reliability, three trained researchers abstracted data from 20 patients and compared results five charts at a time, adjudicating any discrepancies. Decisions were logged in an operational guide to support consistency over time. The chart abstractors had high inter-rater reliability; the raters matched 100% percent by the final set (kappa = 1.0). Then, they conducted the remaining chart reviews individually with frequent discussion to clarify uncertainties and prevent drift.

### Qualitative analysis of goals-of-care documentation

#### Phase 1

To be considered goals-of-care documentation, a note had to include discussion of: (a) prognosis or illness understanding; and (b) goals or treatment options. Full text of all goals-of-care discussions were further characterized by: cancer stage understanding or curability; prognosis: life expectancy; prognosis: what will happen in the future; overall goal of care; cancer treatment options; code status or life-sustaining treatments; hospice; and emotional or spiritual needs [5]. The source of each goals-of-care documentation was collected, including whether it was documented in the formal, health system-wide, preexisting ACP Note template that included the following fields: patient has decisional capacity [yes/no]; surrogate decision maker [yes/no]; health care power of attorney [yes/no], name and contact information of surrogate; discussion participants; communication of medical status/prognosis; communication of treatment options/goals; and treatment decisions. Best practices for all clinicians included documenting goals-of-care conversations and decision making in a separate, easy to locate, place in the EHR such as an ACP Note. We compared best practices within and outside of ACP Notes.

#### Phase 2

We used a template analysis approach to qualitative description, which is a qualitative method that incorporates deductive interpretations of data based on existing conceptual frameworks [15]. Our coding schema was based on existing frameworks for high-quality communication: Braddock et al.’s Informed Decision Making Criteria and Ariadne Labs’ Serious Illness Conversation Guide [6, 16]. Best-practice communication included [1] discussion of prognosis and cancer stage understanding [2]; discussion of broad goals of care and specific treatment options; and [3] documentation of decision making. Through a series of investigator meetings, we developed consensus on the coding framework (NCE, KLW, GSW, LCH), which we modified iteratively until we reached thematic saturation. Two coders (NCE, KLW) were trained on the coding framework. Together, both coders reviewed all goals-of-care discussions and consensus coded using the final coding framework and adjudicated discrepancies with a physician-investigator (LCH). We used ATLAS.ti (Scientific Software Development GmbH) for qualitative data management.

### Quantitative analysis

We conducted univariate and bivariate statistics for demographic and clinical characteristics, dichotomized by whether or not patients had a documented goals-of-care discussion. We also conducted bivariate (t-test) analysis of the content of goals-of-care documentation when that documentation occurred in an ACP Note template versus elsewhere in the EHR.

## Results

### Participants

We identified 492 eligible patients with Stage IV cancer, 206 (42%) of whom had any documented goals-of-care discussion. Forty-seven (9.6%) had a communication barrier due to confusion and sedation, or dementia, and included surrogate decision makers (Table 1).

**Table 1:** Characteristics of patients with Stage IV cancer and acute illness hospitalization, dichotomized by presence of EHR-documented goals-of-care discussion

Characteristic n(%)	Total ***n*** = 492	Documented Goals-of-Care Discussion ***n*** = 206	No Documented Goals-of-Care Discussion ***n*** = 286	***p***
**Age,** mean (range)	60.2 (21–93)	60.7 (21–90)	59.9 (22–93)	0.482
**Female sex**	252 (51)	103 (50)	149 (52)	0.646
**Race/Ethnicity**				
Caucasian	303 (62)	122 (60)	181 (64)	0.512
African American	143 (29)	59 (29)	84 (30)	
Latino/Hispanic	23 (5)	13 (6)	10 (4)	
Asian	9 (2)	5 (2)	4 (1)	
Other	8 (2)	4 (2)	4 (1)	
**Primary cancer diagnosis**				
Gastrointestinal	112 (23)	61 (30)	51 (18)	0.009
Genitourinary	91 (19)	40 (19)	51 (18)	
Breast	87 (18)	25 (12)	62 (22)	
Lung	80 (16)	38 (18)	42 (15)	
Head and Neck	53 (11)	17 (8)	36 (13)	
Melanoma	33 (7)	10 (5)	23 (8)	
Neuro	13 (3)	5 (2)	8 (3)	
Other	23 (5)	10 (5)	13 (5)	
**Primary reason for hospitalization**				
Acute medical illness	267 (54)	105 (51)	162 (57)	0.525
Uncontrolled symptoms	168 (34)	73 (35)	95 (33)	
Failure to thrive	35 (7)	18 (9)	17 (6)	
Acute confusion/ delirium	21 (4)	10 (5)	11 (4)	
Other	1 (0.2)	0 (0)	1 (0.4)	
Comorbidity (CCI)**, mean (range)	6.9 (2–15)	6.9 (2–13)	6.8 (2–15)	0.318
**Nutritional insufficiency diagnosed within 3 days of hospitalization**	226 (46)	115 (56)	111 (39)	< 0.001
Malnutrition	171 (35)	91 (44)	80 (28)	< 0.001
Unplanned weight loss	155 (32)	78 (38)	77 (27)	0.010
Failure to thrive	60 (12)	36 (17)	24 (8)	0.002
Cachexia	40 (8)	31 (15)	9 (3)	< 0.001
Serum albumin level within 3 days of hospitalization*, mean g/dL (range)	3.2 (1.2–5.4)	3.1 (1.2–4.8)	3.3 (1.7–5.4)	< 0.001
**Length of stay, days** mean (range)	5.2 (0–57)	6.2 (0–48)	4.5 (0–57)	0.002
**Deaths**	156 (32)	110 (53)	46 (16)	< 0.001
Survival, days median (range)	28 (3–60)	27 (3–57)	33 (3–60)	0.152

*missing for 14% of patients

**CCI = Charleson Comorbidity Index; range 0–37; higher scores indicate higher disease burden

#### Elements of goals-of-care documentation

Among the 206 patients who had documented goals-of-care discussions, clinicians frequently documented overall prognosis, lack of curability, or cancer stage in communication about illness understanding (89%) but less commonly addressed life expectancy (22%) or future illness trajectory (10%).

Broad goals (e.g., prolong life, support function, improve comfort) and values were documented for 83% of patients. At least one treatment preference was assessed for 98% of patients; options discussed included cancer treatment (62%), hospice (62%), resuscitation (51%), or intensive care (38%). Within this sample, 40% of clinicians documented making a recommendation as part of the discussion, and 15% documented the ways they addressed spiritual or emotional needs during communication.

A clear treatment decision was documented for 82% of patients (Table 2).

**Table 2:** Qualitative coding framework, themes, and quantitative results

THEME n(%)	Total N = 206	Documented in ACP Note ***N*** = 84	Not Documented in ACP Note ***N*** = 122	***p***-value
**Section 1: Documentation of Prognosis & Disease Stage Understanding**				
Assess illness understanding	183 (89)	76 (90)	107 (88)	0.54
Life expectancy	46 (22)	19 (23)	27 (22)	0.93
What will happen in the future	21 (10)	7 (8)	14 (11)	0.47
**Section 2: Documentation of Decision Alternatives, including Broad Goals of Care & Treatment Options**				
Explore goals and values	171 (83)	76 (90)	95 (78)	0.02*
Broad goals of care (includes longevity, function, comfort) & QoL	141 (68)	67 (80)	74 (61)	< 0.01*
Personal Goals (e.g., family, location/home, events)	87 (42)	36 (43)	51 (42)	0.88
Tradeoffs (including risks/benefits)	58 (28)	20 (24)	38 (31)	0.25
Explore patient treatment preferences	201 (98)	81 (96)	120 (98)	0.38
Hospice	128 (62)	52 (62)	76 (62)	0.96
Cancer treatment	127 (62)	48 (57)	79 (65)	0.27
Code status	105 (51)	64 (76)	41 (34)	< 0.01*
ICU/life support/machines	79 (38)	37 (44)	42 (34)	0.16
Surrogate decision making	10 (5)	7 (8)	3 (2)	0.05
Discuss uncertainty (medical)	8 (4)	3 (4)	5 (4)	0.85
Make recommendations/treatment no longer an option	82 (40)	34 (40)	4 (39)	0.87
Address emotional/spiritual needs	31 (15)	6 (7)	27 (22)	< 0.01*
**Section 3: Documentation of Decision Making**				
Document a clear decision/plan	170 (83)	70 (83)	100 (82)	0.80
Document ongoing decision making process	46 (22)	24 (29)	22 (18)	0.07
Provide information about options with unclear next steps; no documentation of shared decision making	8 (4)	2 (2)	6 (5)	0.36

#### Qualitative content of goals-of-care documentation

##### Domain 1: documentation of Prognosis & Cancer Stage Understanding

In this domain, communication about prognosis was commonly documented and often included language about cancer stage and lack of potential for cure (*n* = 183, 89%). One clinician documented, “*Patient hopeful, but aware that her cancer is not curable.*” Less common was description of prognosis in terms of life expectancy, (*n* = 46, 22%) as in this example: “*We reviewed poor prognosis measured in days to weeks. [ …*] *She and her daughters understand.*” Prognosis documentation also rarely described communication on what would happen in the future, (*n* = 21, 10%); as one clinician noted, “*She understands that someday radiation and chemotherapy will not be able to fix her and that she will succumb to her cancer.*”

##### Domain 2: documentation of decision alternatives, including broad goals of Care & Treatment Options

**Category 1, goals and values:** Clinicians frequently documented exploration of goals and values (*n* = 171, 83%). This aspect of documentation most frequently took the form of exploring goals of care broadly (*n* = 141, 68%), as in this example: “*He doesn’t want her to suffer unnecessarily but still believes she is strong and has the will to live. He is open to hospice at home if she survives to discharge, but not ready to consider comfort care.*” Documentation of goals and values also included the more specific sub-categories: personal goals (*n* = 87, 42%), such as this conversation about a time-limited goal, “*He would like to live long enough to see his grandchild born in December and articulates his desire to continue chemotherapy if he could meet this goal.*” Goals were also specified in terms of their corresponding tradeoffs (*n* = 58, 28%), with different values-based conclusions, as was the case with these two different patients:

*In his mind, he is not sure if that extra time would be worth the suffering, especially since he is at peace with his relationship with God and knows that he will be saved.*

*He acknowledges the reality that treatments carry risks and side effects and may cause discomfort. He is willing to accept discomfort in order to extend his life.*

**Category 2, treatment preferences:** Clinicians documented exploration of patients’ treatment preferences in nearly all cases of documented goals-of-care discussions (*n* = 201, 98%) for several treatment types (sub-categories) including:

Hospice (128, 62%): In this example quote, the clinician notes examination of the intersection of the patient’s priorities with treatment decisions,

*She expressed that she had been worried about being away from her home and being unable to communicate with non-Spanish speakers. She was delighted to learn that home hospice services for Spanish speakers can be provided though most agencies in her county. She requested to be discharged home with home hospice.*

Cancer treatment (*n* = 127, 62%): In the example quote here, the clinician documents a discussion of a pause in chemotherapy to consider options, and contract further chemotherapy again best supportive care,

*Daughter states that given how poorly he’s been feeling, he had been thinking about stopping further chemotherapy. We discussed that we will take one week off therapy to allow more time for recovery from chemo. If he still feels poorly, proceeding with best supportive care would be reasonable.*

Code status (*n* = 105, 51%): Here, we see an example of a more robust discussion (compared to simply listing code status) that acknowledges malleability of goals and preferences over time, “*Patient wants to remain Full Code for now, but acknowledges she might consider DNR as she nears the end of life*.”

ICU or other life support (*n* = 79, 38%): Here, the clinician’s note shares information about future life support treatment possibilities, “*We discussed my concerns that if her hypoxia worsened as a result of her cancer, intubating her and maintaining her on life support would be challenging as there would no one intervention to reverse her hypoxia and allow her to return to normal living.*”

Surrogate decision making was explicitly discussed in 5% (*n* = 10) of goals-of-care documentation, excluding a simple list of the surrogate decision maker name, relationship, and contact information. These conversations often took the form of establishing the decision maker,

*She is able to clearly answer questions when asked and has capacity to make decisions, but withdraws from the conversation when addressing goals of care. She would like her family to help make the decisions for her and trusts that they will have her best interest in mind.*

**Category 3, uncertainty:** Clinicians infrequently documented discussion of uncertainty (*n* = 8, 4%). When uncertainty was discussed, it took the form of assessing the nature of specific elements of the disease or complications, for example,

*We discussed that it will be important to determine the etiology of his acute renal failure. If no intervention, could potentially progress from a renal failure perspective, which could be life limiting on a shorter time-frame than his prostate cancer.*

**Category 4, make recommendations:** Including notes that indicated treatment was no longer an option, clinicians documented discussion of recommendations in 82 (*n* = 40%) cases. Recommendations were varied, from hospice,

*In-patient hospice recommended due to high symptom burden (mucous plugs and desaturations requiring deep suctioning, pain management) and frequent interventions (tube feeds, medications),*

to code status and specific treatments,

*I gave him my opinion that ventilator support and cardiac resuscitation as medical interventions were unlikely to make him live longer, and might impose significant suffering, although I certainly think it is reasonable to provide interventions short of this to cover all reversible causes.*

Goals-of-care documentation infrequently addressed spiritual and emotional needs (*n* = 31, 15%). Information of this nature tended to demonstrate information sharing with future clinicians, for example, “*She states she is Muslim and declines chaplain services at this time. She reports continued communication with her therapist during her stay, which has been very helpful,*” and, “*Counseling and support with patient. Discussion of diagnosis, feelings of anger, fear and anxiety. Acknowledgement of loss of ability to drive, fish and to do the things he enjoys.*”

##### Domain 3: documentation of decision making outcomes

Decisions were documented in three ways. Categories were not mutually exclusive within each case, or with respect to different decisions.

##### Category 1

Documentation included a clear decision or plan in 170 cases (82%). These notes included clear next-steps, such as, “*We will continue the aggressive measures we have already embarked on but will not intubate or resuscitate if she continues to decline.*”

##### Category 2

Documentation included note of ongoing decision-making processes in 46 cases (22%), including the patient or family’s desire to continue the discussion with each other of the clinician at a later time,

*Best supportive care would be a very reasonable treatment decision, and indeed would probably be what I would recommend if the patient were my family member. His family understands this discussion, particularly since he has experienced significant toxicity with more benign medications that he has been on for long periods of time like his antiepileptics and antihypertensives. They would like to discuss over the holiday and follow up after molecular testing returns.*

##### Category 3

Clinicians documented that they provided information about options with unclear next steps, or provided no documentation of shared decision making (*n* = 8, 4%). For example, “*She articulates a desire to explore the option of a large complicated operation vs hospice. [ …] She is frequently tearful on exam.*”

#### Associations between goals-of-care documentation and ACP note template

Among patients with goals-of-care documentation (*n* = 206), that documentation was in an ACP Note in 127 (41%). On average, documentation of goals-of-care discussions in ACP Notes included 8.7 (SD 1.98) categories, whereas documentation outside ACP Notes included 8.0 (SD 1.97) categories (*p* = 0.01).

Compared to documentation outside of ACP Notes, goals-of-care discussions that were documented in ACP Notes more frequently included exploration of goals and values (78% vs. 90%, *p* = 0.02), particularly about broad goals of care (including longevity, function, comfort, and quality of life; 61% vs. 80%, *p* < 0.01). Relatedly, discussion of code status was more often documented in ACP Notes than not (76% vs. 34%; p < 0.01). Emotional and spiritual needs were more frequently included in notes documented outside ACP Notes (22% vs. 7%; *p* < 0.01). There were no other significant differences in the content of goals-of-care documentation whether in ACP Notes or otherwise (Table 2).

## Discussion

We have developed and applied an operational definition of clinical documentation of goals-of-care discussions, grounded in published conceptual frameworks of high-quality communication [5, 6, 16]. Fewer than half of patients with Stage IV cancer and acute hospitalization had a documented goals-of-care discussion. Among documented goals-of-care discussions, almost all explored patient treatment preferences and many explicitly addressed patient goals and values. Documentation of prognostic communication and attention to patient spiritual and emotional needs was less common.

Documentation of goals-of-care discussions is an important component of high quality goals-of-care communication skills. High-quality documentation is necessary (though likely not sufficient, alone) to ensure that other clinicians can understand patient values, preferences and honor current treatment decisions, thus supporting clinical aspects of goal-concordant care [17]. However, we found very little guidance for frameworks and recommendations for goals-of-care documentation practices, instead we relied on best-practices for communication, an imperfect proxy [6, 16].

One potential strategy to improve goals-of-care documentation is use of an ACP Note template. We found that use of this tool promoted more complete and detailed documentation. Templated EHR notes may also improve clinician-clinician communication in other ways, because [1]: the notes can then be systematically searched and reviewed for real-time decision making, and [2] templates include structure to prompt inclusion of important content, thus increasing the likelihood of including all information essential to decision making [18]. For example, because clinicians’ understanding of emotional and spiritual concerns is important to decision making, including explicit space for such information could enhance documentation.

The purpose of documentation is to tell other clinicians the current preference-driven treatment plan, transmit an understanding of the patients’ goals and preferences, indicate current uncertainty or need for future decision-making, and other essential points to good communication with a specific patient (e.g., noting complex family dynamics or application of religious or spiritual beliefs to medical decision making) [19]. These nuances can have large consequences on future communication and care transitions. If the next clinician cannot determine next steps, the current state of the decision-making process, or even find the note, it will likely negatively affect patient care, particularly when a patient’s decisions on not consistent with clinical defaults (generally for life prolongation). This is manifested in evidence that documentation of decisions for patients with cancer and other serious illness occurs infrequently, and often very near the end of life, potentially limiting access to treatments consistent with patient goals, including hospice [20–22].

This study was conducted among patients with Stage IV solid-tumor cancer, limiting its generalizability to decision-making in patients with earlier-stage disease. This study was also conducted at a single public hospital affiliated with an NCI Comprehensive Cancer Center; therefore the patient population is quite broad, though findings may not generalize to smaller or non-academic centers. Although ACP Note templates vary, our conclusions may still be applicable to different settings in terms of similar tools.

We set a relatively high standard for inclusion of goals-of-care documentation in this analysis, and focused on goals-of-care rather than all advance care planning, unlike, for example, National Quality Forum (NQF) metrics for documentation of treatment preferences, that are generally more inclusive [23]. Considering low rates of some arguably important pieces of information, documentation corresponding to goals-of-care conversations can be more robust.

## Conclusion

Just as there have been expert-defined and empirically-informed best practice guidelines for in-person communication with patients and their families, additional guidelines should define best-practices for documentation to maximize clinician-clinician communication without inducing undue documentation burden [8]. Additional empiric work can link documentation practices and patterns to whether patients receive goal-concordant care.

## Data Availability

The datasets used and analysed during the current study are available from the corresponding author on reasonable request.

## Abbreviations

EHR: electronic health record
ACP: Advance Care Planning
UNC: University of North Carolina
NQF: National Quality Forum
